# From prescriptions to drug use periods - things to notice

**DOI:** 10.1186/1756-0500-7-796

**Published:** 2014-11-14

**Authors:** Antti Tanskanen, Heidi Taipale, Marjaana Koponen, Anna-Maija Tolppanen, Sirpa Hartikainen, Riitta Ahonen, Jari Tiihonen

**Affiliations:** Department of Clinical Neuroscience, Karolinska Institutet, Stockholm, Sweden; National Institute for Health and Welfare, Helsinki, Finland; Department of Forensic Psychiatry, Niuvanniemi Hospital, University of Eastern Finland, Kuopio, Finland; Kuopio Research Centre of Geriatric Care, University of Eastern Finland, Kuopio, Finland; School of Pharmacy, University of Eastern Finland, Kuopio, Finland; Research Centre for Comparative Effectiveness and Patient Safety (RECEPS), University of Eastern Finland, Kuopio, Finland

**Keywords:** Prescription register, Modeling, Drug utilization, Pharmacoepidemiology

## Abstract

**Background:**

Electronic prescription registers provide a vast data source for pharmacoepidemiological research. Prescriptions as such are not suitable for all research purposes; e.g., studying concurrent use of different drugs or adverse drug events during current use. For those purposes, data on dispensed prescriptions needs to be transformed to periods of drug use.

**Methods:**

We used 3,828,292 dispensed prescriptions claimed between 1 January 2002 and 31 December 2009 for 28,093 persons with Alzheimer’s disease. Examples of drug use histories are presented to discuss different aspects that should be noticed when using register-based data consisting of drug purchases.

**Results:**

There is no simple method for correctly transforming dispensed prescriptions to periods of drug use that is usable for all drugs and drug users. Fixed assumptions of daily dose (in defined daily doses, tablets or other units) and fixed time windows should be used with caution and adjusted for different drug use patterns.

**Conclusions:**

We recommend that when transforming prescription drug purchases to drug use periods personal dose, purchasing pattern and other behavioral differences between patients should be taken into account.

## Introduction

Electronic prescription registers have been used in medical research since the early 1990’s [1–4] although the history of prescription databases began much earlier [5, 6]. Nationwide prescription data from Nordic countries have generated several publications; e.g., a recent review lists 515 studies published between 2005 and 2010 [7]. At first, prescription registers were mainly used to collect drug use statistics and drug utilization data, mostly to determine adherence [1, 8, 9]. The possibility of linking a person’s prescriptions and outcome data with hospital discharge or death registers, for example, have enabled researchers to conduct nationwide studies to measure effectiveness and adverse events, such as morbidity or mortality.

Prescription registers contain information on the prescriber, patient, drug (ATC classification, package information), time of dispensing and possible dosage instructions in free text format (e.g., details of Nordic data [10]). Prescription data are not, however, directly useful for many research questions; e.g., in the study of outcomes related to current drug use, exposure status at the index date of the outcome must be determined. Furthermore, dosages must be defined if the dose–response relationship is a matter of interest. Both exposure periods and dosages can be estimated from prescription data by modeling individual drug use.

Prescription registers contain dosage information via Defined Daily Dose (DDD), which is the assumed average maintenance dose per day for a drug that is used for its main indication in adults [11]. The DDD is an effective rough measure for comparing the consumption of different drugs. However, the assumption of one DDD as a standard dose can be erroneous, as DDD is the dose used in monotherapy for a specific indication. Moreover, the actual dosage may vary widely between different indications or patient groups, such as children and older people. The DDD is often a compromise of doses used in several countries [11]. However, DDD may be used as a universal measure of the purchased amount of drug, rather than physical measures such as milligrams or milliliters. Eventually, the purchased amount of drug, which is often reported as DDD in prescription register data, can be translated into other units (like milligrams) by using the ATC-DDD index. Thus, DDD is a practical measure to use when translating data from prescription registers to periods of drug use, if its limitations are appropriately accounted for.

The number of dispensed units, such as tablets, can also be used to estimate drug use periods. The main problem with this information is that the number of tablets taken per day may vary between different strengths of the same drug. There may also be large variations in dose per day for different patients using the same drug. In contrast, DDD is independent of packet size and strength of the drug (e.g., ten 10 mg tablets are equal to five 20 mg tablets of the same formulation).

The aims of this paper is to present some perspectives on how to translate prescription data to periods of drug use, and to introduce critical elements that should be considered when constructing drug use periods from prescription register data.

## Material and methods

### Data source

The examples in this paper derive from the MEDALZ-2005 cohort prescription data, which included all community-dwelling persons with a verified diagnosis of Alzheimer’s disease residing in Finland on 31 December 2005 (n =28,093) [12] . Persons with Alzheimer’s disease have been identified from special reimbursement register. This register includes data on entitlement to special reimbursement due to several chronic diseases (including Alzheimer’s disease, asthma and diabetes) that have been diagnosed by a physician. Data of MEDALZ-2005 cohort has been linked to several nationwide registers including prescription register, special reimbursement register, hospital discharge register (Hilmo) and register of care at social institutions. Altogether 3,828,292 prescriptions were claimed between 1 January 2002 and 31 December 2009 (on average 136 prescriptions per person). In Finland, drugs may be dispensed for a maximum of three months’ treatment period at a time. The Finnish Prescription Register does not cover non-reimbursable drugs, over-the-counter drugs or drugs used in hospitals and nursing homes.

### Calculations

DDD per day values were calculated by sliding temporal averages to smoothen variations in purchase histories. This approach takes into account potential changes in the patient’s personal drug stock at the time of the next prescription purchase. For each purchase, a sliding temporal average for dose (DDDAVG_i_) was calculated as follows:


where DDD_i_ is the DDD amount purchased at time i, and T_i_ is the amount of days between purchase i and i + 1. We weighed consecutive drug purchases and times with weights 1, 4 and 1 for i-1, i and i + 1, respectively. For each person’s first purchase of each ATC-class, drug and time weights were 5 for i, 1 for i + 1, and for last purchase 1 for i-1 and 5 for i. For the last purchase i T_i_ is calculated from the previous purchase assuming the same DDD per day value as previous purchase, T_i_ = DDD_i_/(DDD_i-1_/T_i-1_). This assumption that dose does not change is used for convenience. The weights were selected to give moderate smoothening over time. Average DDDs per day were calculated only when there were at least three purchases of the same ATC code for one person. Hospital days were excluded (i.e., days in hospital have been subtracted from T), as drugs are provided by the hospital during hospital stays, and this information is not entered in the prescription register. These calculations were made for all drugs used by the cohort and recorded in the prescription register.

We compare three methods for converting purchases to drug use periods: Firstly one DDD per day rule, in which persons are assumed to take drug one DDD per day, i.e. a package containing 30 DDD equivalents lasts 30 days [13]. Secondly we describe one tablet per day rule, which assumes that drug is consumed 1 tablet (unit) per day [14, 15]. Thus, one package of 30 tablets would last 30 days. Thirdly, fixed time window methods (time since last purchase, calendar time or grace periods) are described. Fixed time windows may define a time period from the last purchase when the next purchase must take place if drug use is continuous [16]. For example, the next purchase must take place within 90 days from the last purchase or else drug use is considered discontinued. Fixed time windows are also used in terms of calendar time, where current drug use is determined by purchases taking place during fixed calendar time periods, for example in three months [17]. In addition, fixed time windows are used as grace periods, when fixed time is added to drug use time calculated from purchased amount and/or dose [18].

## Results and discussion

### One DDD per day rule

The average DDD per day typically varied between 0.5 and 2 for many of the commonly used drugs in the MEDALZ-2005 data. Some drugs were used at a lower dose; e.g., antipsychotics (ATC class N05A), where mean DDD per day dose for quetiapine (N05AH04) was 0.25 (N of purchases = 42,950; S.D. 0.44) and for risperidone (N05AX08) 0.21 (N of purchases = 53,864; S.D. 0.39). These drugs were widely used among MEDALZ-2005 participants with AD, with over 40,000 purchases of both drugs. In such cases, the assumption of one DDD per day would clearly underestimate the length of exposure period and lead to an erroneous conclusion of several short recurrent drug use periods for most patients.

**Figure 1:**
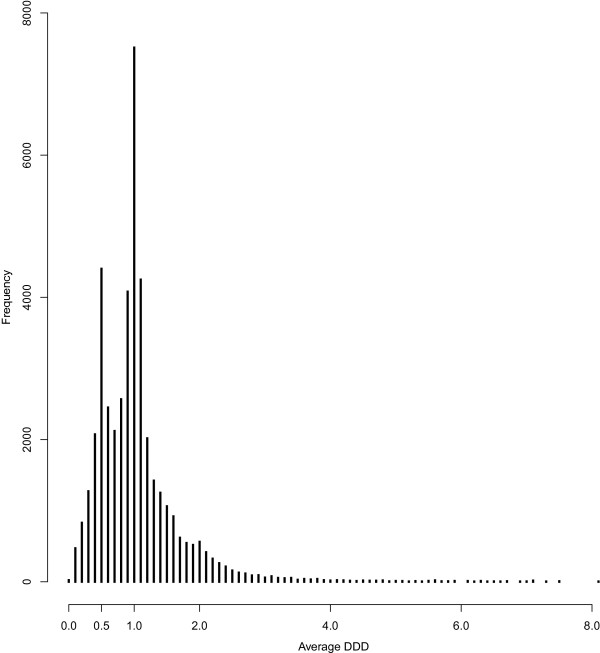
**DDD**_**AVG**_ **distribution of temazepam (N05CD07), two most commonly used doses were 0.5 and 1.0 DDD per day.**

In MEDALZ-2005 data, temazepam (N05CD07) and zopiclone (N05CF01) typically averaged 1 DDD per day; 1.10 (N = 43,664; S.D. 2.07) and 1.18 (N = 59,728; S.D. 1.86), respectively. In both cases, however, the standard deviation reveals a high variation in DDD per day values. Figure 1 describes distribution of average DDD per day doses for temazepam used by MEDALZ-2005 participants. There are two clusters of DDD values; one at 0.5 DDDs per day and the other at 1.0 DDDs per day. Some patients use temazepam at a higher dosage, approximately 1.5-2.0 DDDs per day and, thus, distribution spreads to higher values and the average is greater than 1 DDD. Assumptions such as 1 DDD per day, or any other fixed dose assumption, may lead to biased estimates and only individually calculated dose estimates will be appropriate in such cases.

**Figure 2:**
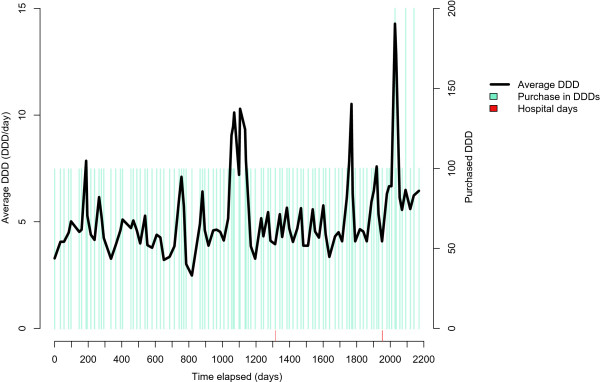
**A case history with a high number of purchases (n = 101) and high DDD per day dose (4–6) of lactulose (A06AD11), over six years.**

Figure 2 illustrates a purchase history for a patient using lactulose (A06AD11, laxative) over 2,000 days (about 5.5 years). The purchased amount is 100 DDDs at one time until the end of follow-up, when 200 DDDs are purchased at a time. DDD per day varies between 4 and 6, except in the middle of the drug use period (after 1000 days), when it increases up to 10 DDDs. The time between purchases was typically between 20–30 days. This example presents an unusually large variation in dosage, possibly due to patient’s condition, and the assumption of 1 DDD per day is clearly not valid. Very high values for DDDs per day typically result from refills that occur shortly after a previous purchase, and are not necessarily real dosages. For this population, dosages of many drugs are below one DDD per day due to advanced ages, high prevalence of renal dysfunction and several comorbidities and concomitant use of multiple drugs for same disease.

### Use of package size and strength – the one tablet per day rule

The prescribed number of tablets per day is a useful measure of drug exposure, but this information is difficult to extract from prescription registers, especially when the written dosage instructions are complicated or missing. In Nordic countries, Nordic Article numbers (vnr-numbers) are used to identify the drug package (i.e., drug substance, manufacturer, number of drug units, strength and dosage form) [19]. Almost all drugs are sold in different package sizes, for example 30 and 100 tablets representing treatment periods of one and three months, respectively. Drugs that are mainly used occasionally, for example non-steroidal anti-inflammatory drugs (NSAIDs), are often marketed with various package sizes.

When package information is available and coded with package identification numbers (such as vnr-numbers), such information can be utilized in large datasets to determine typical refill lengths for each package. Refill length refers to the interval between two purchases. Refill length describes how long the package lasts and, thus, the typical daily dose. Typical refill lengths for each package are useful when there is not enough information to evaluate drug use period like when a person who has purchased the drug only once during the follow-up. Douglas and Smeeth used package information in their study to fill gaps in purchase information [20].

**Figure 3:**
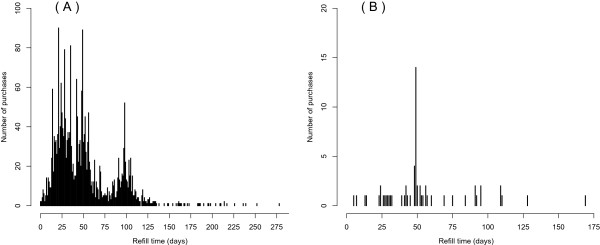
**Distribution of refill time lengths for a single package of 25 mg quetiapine, package size 100 tablets, Nordic article number 075533 in (A), and for 200 mg quetiapine, package size 100 tablets, Nordic article number 075551 in (B).**

Quetiapine (N05AH04) is presented as an example of different drug use patterns with different drug strengths (Figure 3A and B). This example includes quetiapine in 25 mg tablets (Vnr 075533, 25 mg, 100 tablets) and 200 mg tablets (Vnr 075551, quetiapine 200 mg, 100 tablets). In Figure 3A, peaks describing the most common refill lengths of quetiapine 25 mg tablets are 20, 25, 33, 50 or 100 days per package. The peak at 20 days indicates that a group of individuals purchased 100 tablets between 20 days interval and, thus, used 5 tablets a day. Quetiapine 25 mg tablets are therefore used at a rate of one to five tablets per day in this cohort. Long refill lengths indicate some restarts in treatment, or possible instructions to take tablets as needed (e.g., in addition to regular use of another tablet strength). Quetiapine 200 milligram tablets (Figure 3B) are typically used at a rate of two tablets per day (peak at 50 days), and this dosage is equivalent to one DDD per day. The 200 mg quetiapine product is infrequently used within the MEDALZ-2005 cohort (61 purchases), compared to the 25 mg product (3,815 purchases). The distributions of refill times presented here are limited to this population, and are not transferrable to other populations, especially younger individuals who may have a different indication of use and, consequently, a different dose. Polypharmacy may also explain low doses in some cases. Examples above show how difficult it is to use any exact number of tablets per day rules to estimate drug use periods.

### Regularity of purchases

**Figure 4:**
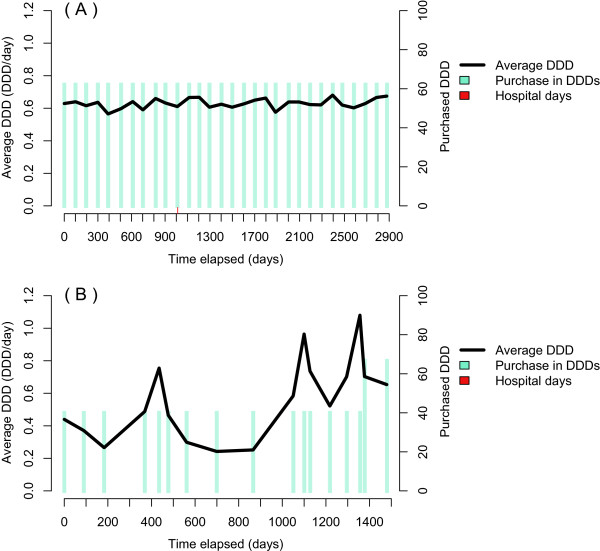
**Two different purchase patterns of metoprolol (C07AB02); a regular in (A) and an irregular in (B) with variations in both purchased amount and intervals between purchases.**

Patients display varying patterns of purchases from the pharmacy, as some visit pharmacy at regular intervals and the same amount is dispensed each time. Often this idiosyncratic style is stable and could be used in modeling periods of drug use. Figure 4A presents a patient who bought 60 DDDs of metoprolol (C07AB02, beta blocker), at regular time intervals of 90–100 days. Hardly any stockpiling could be expected with this purchase history, and the dosage is expected to be between 0.6 and 0.7 DDDs per day. There are no signs of titration periods in this purchase history example.

An irregular purchase history of warfarin, an anticoagulant, shows how purchases can be clustered in time (Figure 4B). At the beginning, the patient purchases packages containing 40 DDDs of warfarin for almost four years, with 21–184 days between purchases. In this example, the last two purchases of warfarin included 67 DDDs each, which may indicate a dosage change. Some stockpiling may have also occurred between days 370–560 (i.e., frequent purchases of warfarin). This patient had probably used warfarin as prescribed, but with rigid rules for fixed amount of DDD per day (here the average is 0.45 DDD per day), or fixed amounts of tablets per day, the patient would have periods of both high and low adherence.

### Stockpiling

Stockpiling is a fairly common phenomenon with drug purchases. Sometimes stock may be large, especially during holiday seasons and periods of travel. Although reimbursement regulations generally try to limit stockpiling, such regulations may also induce seasonal variation; e.g., by exceeding the annual excess costs at the end of the calendar year. During week 51 before Christmas in 2007, for example, 28,093 cases from the MEDALZ-2005 data had purchased 769,501 DDDs of drugs, whereas the second week in January of 2008, only 497,626 DDDs were purchased. Both weeks had the same amount of working days and thus, equal access to pharmacies. Thus, stockpiling helps to explain at least some irregularity in purchases.

Table 1 shows a purchase history for combination of losartan and diuretic (C09DA01, antihypertensive drug). A patient uses losartan with 1.0 DDD per day calculated from 27 purchases, over 2,747 days (ca. 7.5 years), assuming no stock in the beginning or at the end of study period. At day 271, the patient bought two packets of drug and slowly used the stock so that on day 1,675 (ca. 4.6 years) the stock was negative. This indicates the presence of some missed tablets (i.e., not perfect adherence) or some stock at the beginning of the follow-up period. Because the cumulative stock varied around zero, between −15 and 15 DDD during the rest of follow-up, it likely that there were still some tablets left by day 1,675, and possibly some stock at the beginning of the follow-up.

**Table 1:** **A purchase history of combination of losartan and diuretic (C09DA01) is presented as an example of stockpiling**

Time from the first purchase in days	Time from the previous purchase in days	DDD purchased	Assumed refill length with 1.0 DDD per day dose in days	Units stockpiled during the current purchase in DDD	Cumulative units stockpiled from first purchase in DDD
0	0	98,00	98		
84	84	98,00	98	14	14
187	103	98,00	98	−5	9
271	84	196,00	196	112	121
364	93	98,00	98	5	127
435	71	98,00	98	27	154
539	104	98,00	98	−6	148
679	140	98,00	98	−42	106
746	67	98,00	98	31	137
868	122	98,00	98	−24	113
967	99	98,00	98	−1	112
1078	111	98,00	98	−13	99
1149	71	98,00	98	27	126
1359	210	98,00	98	−112	14
1400	41	98,00	98	57	72
1514	114	98,00	98	−16	56
1675	161	98,00	98	−63	−7
1774	99	98,00	98	−1	−8
1879	105	98,00	98	−7	−15
1963	84	98,00	98	14	−1
2061	98	98,00	98	0	−1
2145	84	98,00	98	14	13
2271	126	98,00	98	−28	−15
2341	70	98,00	98	28	14
2439	98	98,00	98	0	14
2536	97	98,00	98	1	15
2649	113	98,00	98	−15	0
2747	98	98,00	98	0	0

In this example, combination of losartan and diuretic is used on average 1.0 DDDs per day (i.e., 1 tablet per day).

The purchase on day 271 created a stock that remained until the purchase on day 1,359. Otherwise this patient had quite a regular purchase pattern.

### Changing dosage

In long-term use, drug dosage may vary due to disease progression, changes in lifestyle and nutritional factors (e.g., with lipid modifying agents), or drug tolerance (e.g., with strong opioids). Changes may include gradually increasing dose, or medically ordered changes in doses according to clinical testing (e.g., with warfarin and antidiabetics). The reasons for such changes are difficult to assess from the prescription register. In these cases, a particular dose at a specific time point may not be equal to the average dose over the entire period of drug use. To overcome this problem, dosage should be estimated over time with local, limited time windows.

Titration of the appropriate drug dose is sometimes required at the beginning of treatment for certain drugs, and often there is a standard procedure on how to increase the dosage to the effective level. For example, the use of the anti-dementia drug donepezil (N06DA02) typically begins with 5 mg per day, or even a lower dosage of 2.5 mg per day for the first few days or one week. Within two to six months, the dose can be increased to the recommended daily dose of 10 mg per day, if the drug is well tolerated by the patient.

**Figure 5:**
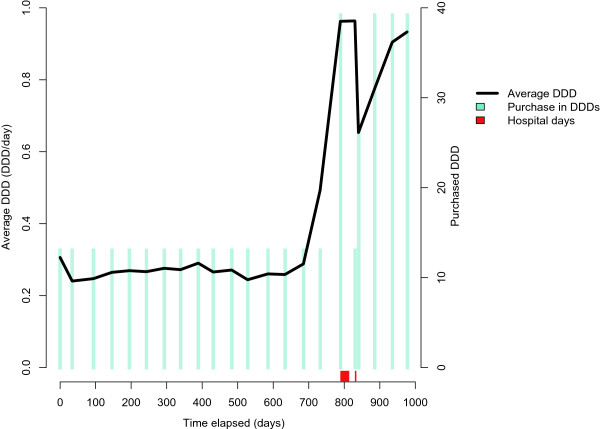
**Increasing dosage and package size of oxycodone (N02AA05) in long-term use.** The dosage is three-fold higher than the initial value by the end of the follow-up after hospital stays marked on timeline.

Figure 5 illustrates an individual’s drug use period of oxycodone (N02AA05, opioid). In this example, oxycodone was used for over three years and the dosage dramatically increased after the second year of use.

### Varying amounts of drug purchased per time

One reason for variations in refill time lengths is due to differing amounts of drug obtained per purchased; e.g., changes in package size, the number of packages and/or strength. Smaller packages may be bought at the beginning of treatment, when it is not certain whether the drug is suitable or even necessary for longer periods. The strength of a drug unit can initially be smaller and when the optimal dose has been found and it may be increased to decrease the amount of tablets taken per day. In addition, the financial situation of the patient may have an impact on the amount of drug purchased each time. Finally, reimbursement regulations also affect refill time lengths, as they are designed to reduce both stockpiling and limit the amount purchased at one time.

**Figure 6:**
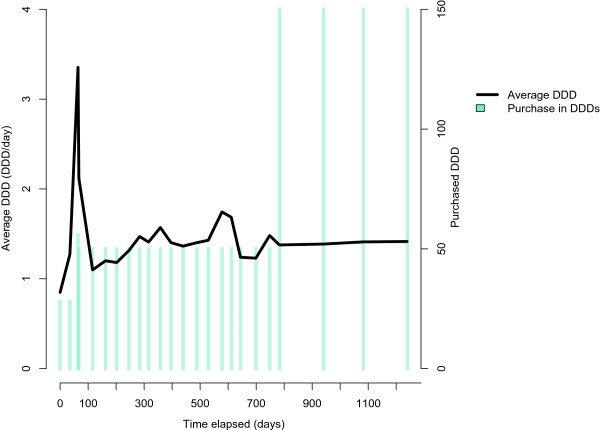
**The amount of DDD purchased; escitalopram (N06AB10).** Refill time length between purchases increased while the dosage remained stable. Patient has no hospital stays.

Figure 6 describes a purchase history of escitalopram (N06AB10), a selective serotonin uptake inhibitor (SSRI). For the first two years, the patient mainly bought 50 DDDs per purchase, and then began purchasing 150 DDDs per purchase. The average DDD per day remained unchanged, but the time between purchases tripled because a larger amount was purchased at a time.

Such purchase histories are complicated and difficult to analyze with confidence. For example, when trying to define the current drug use with the rule whether a purchase occurred during the last 90 days in Figure 6, a person would be categorized as a continuous drug user for the first two years, but subsequently the refill time length increased to 150 days, which suggests 90 days of drug use and 60 days of non-use. Longer fixed times such as “bought in the last 180 days” would also lead to miscalculations, as the previously ended period of drug use would be classified as current drug use. In contrast, shorter fixed times would omit the actual current usage [18]. When drugs can be purchased in different package sizes and strengths, there is no appropriate fixed value for the refill time length. Thus, individual or package-based calculations are required.

It is also possible that ignoring differences in package sizes leads to a bias in terms of drug use by different economic classes. For example, individuals with low income may be more inclined to purchase drugs in smaller packages (i.e., a smaller charge at a time) than persons with middle or higher income.

### Other sources of drugs

**Figure 7:**
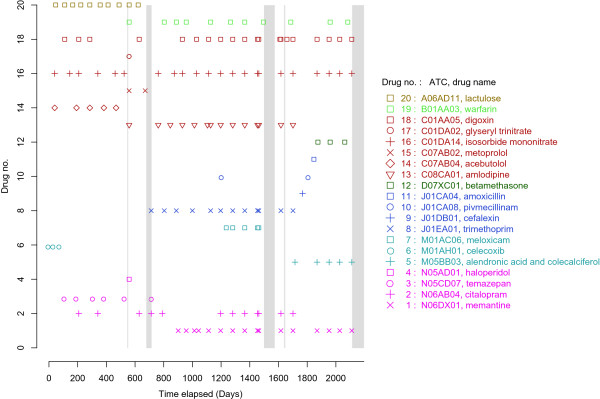
**An individual’s purchase history for about six years, which describes the purchase of 20 different drugs.** Note the long hospital stays between days 679–719, 1506–1574 and 2113–2250 (marked with grey bars). After day 2250, this person has not collected any drugs and resides in nursing home, so no information on drug use is available in the prescription register.

The coverage of prescription registers varies between countries. Thus, patients may get their drugs from other sources than pharmacies, which may not be visible in the prescription register data. In Finland, the drugs used during hospital stays are provided by the hospital. The same applies to several public nursing homes, but nowadays in diminishing proportions. Gaps in the prescription register data may reflect remission or, more likely, long stays in hospital and/or nursing home (Figure 7). Thus, additional information on hospital stays, and time in a nursing home, improves the estimation of true drug use periods. Also, some drugs are available over the counter, in smaller packages or lower strengths, which may bias dosage counts if some of the refills are not readily accessible through registers. Physicians may also give “sample packages” to patients for a few days or weeks of use, thus making the exact starting date unknown. Purchasing drugs while traveling abroad may also lead to an underestimation of drug use. Price differences between countries and variations in the availability of some generic drugs may encourage the purchase of drugs while traveling.

In Finland, some drugs are available over the counter in smaller package sizes and lower strengths, whereas larger packages and higher strengths are only dispensed with a prescription, and typically only these prescription-based packages are reimbursed. Reimbursement and regulatory decisions may change over time, and they are often related to the prices of drugs. Changes in both reimbursement schemes and over-the-counter status introduce variabilities that must be considered when using prescription registers. Such changes may introduce anomalies in the prescription histories, which must be recognized.

## Conclusions

The utilization of prescription register data in pharmacoepidemiological studies requires a simulation of drug purchases by the patient or care-giver. The first step in using such information is to establish how drugs are dispensed and what aspects may affect the purchase behavior. Herein, we have discussed some factors that should be taken into account; i.e., use of the DDD, stockpiling, refill lengths and changing dosages. Some factors, such as regulations on refill times or timing of holiday seasons, may vary in different countries. Still, the same basic principles apply to drug use patterns everywhere; e.g., stockpiling, holidays, and changing doses over long periods of time. Prescription histories are part of a modern human life, yet they cannot be accurately and appropriately modeled with simple assumptions, such as one DDD per day, 1 tablet per day or even by purchase events every 90 days. Previously, most studies used fairly simple models to estimate individual drug use.

We give below a short checklist that should be considered before proceeding with the actual data analysis in a pharmacoepidemiology project with prescription register data. The importance of different aspects varies by country, patient group, ATC class and illness.

- ● Consider whether the assumption of fixed dose (1 DDD per day or 1 tablet per day) is valid for calculating exposure times. This may vary between different drugs (ATC classes).
- ● Assess whether fixed refill times between purchase times are valid. This may need to be checked by ATC class.
- ● Do not underestimate stockpiling. Calculate estimates for stockpiling and pay special attention to holiday seasons.
- ● Use package information if available to make estimates of refill times and dosages.
- ● Written dosage helps, but obtaining this data may be laborious and may not take account on dosage changes (often not written in the prescription if persons has contacted physician by phone).
- ● Be aware of other sources of drugs (hospitals, nursing homes, other countries, over the counter).
- ● Check hospital/nursing home/prison stays for possible drug dispensing gaps in the register data.

Our examples are drawn from an older population with Alzheimer disease, an incurable progressing disease. This population have high rate of polypharmacy, high number of hospital stays and often changing drug regimen. This population is selected as an example because it shows how difficult it is to use simple fixed rules like one DDD per day.

When transforming prescription drug purchases to drug use periods personal dose, purchasing patterns and other behavioral differences between patients should be taken into account.
